# Sustainable Removal of Tolperisone from Waters by Application of Photocatalysis, Nanotechnology, and Chemometrics: Quantification, Environmental Toxicity, and Degradation Optimization

**DOI:** 10.3390/nano12234199

**Published:** 2022-11-25

**Authors:** Szabolcs Bognár, Predrag Putnik, Ivana Maksimović, Branko Velebit, Marina Putnik-Delić, Daniela Šojić Merkulov

**Affiliations:** 1 Department of Chemistry, Biochemistry and Environmental Protection, Faculty of Sciences, University of Novi Sad, Trg Dositeja Obradovića 3, 21000 Novi Sad, Serbia; 2 Department of Food Technology, University North, Trg Dr. Žarka Dolinara 1, 48000 Koprivnica, Croatia; 3 Faculty of Agriculture, University of Novi Sad, Trg Dositeja Obradovića 8, 21000 Novi Sad, Serbia; 4 Institute of Meat Hygiene and Technology, Kaćanskog 13, 11040 Belgrade, Serbia

**Keywords:** photosynthetic pigments, toxicity mechanism, wheat biomass germination, tolperisone, TiO_2_/ZnO

## Abstract

Environmental pollution is an emerging global issue. Heterogenous photocatalytic degradation, which belongs to the advanced oxidation processes, is a promising sustainable technique for the removal of harmful pollutants (e.g., pharmaceuticals) from natural resources (surface and underground waters), as well as wastewaters. In our study, we examined the efficiency of photocatalytic degradation (with TiO_2_ and ZnO as photocatalysts) of tolperisone hydrochloride (TLP) and the effect of TLP and its degradation intermediates on germination, photosynthetic capacity, and biomass production of wheat. According to the UFLC-DAD and LC–ESI–MS results, we found that the complete degradation of TLP can be reached after 60.83 min of UV irradiation using TiO_2_ as a photocatalyst. Furthermore, we determined that germination, biomass production, and chlorophyll b (Chl b) were not related to the percentage of TLP after irradiation. Chlorophyll a (Chl a) (r = −0.61, *p* ≤ 0.05), Chl a+b (r = −0.56, *p* ≤ 0.05), and carotenoid (car) (r = −0.57, *p* ≤ 0.05) were strongly inversely (negatively) correlated with TLP, while Chl a+b/car (r = 0.36, *p* ≤ 0.05) was moderately (positively) related.

## 1. Introduction

In 2020, the world generated 380 billion m^3^ of wastewater, which is 5 times all water that passes yearly over the famous Niagara Falls [1]. In 2012, poor water and sanitation caused USD 0.1 trillion in global economic damages, while unsafe water caused 1.23 million deaths in 2017 [2]. Globally in 2016, the largest wastewater expenditures (49%) included finding solutions to reduce water pollution [3]. Nowadays, environmental pollution originating from pharmaceuticals is a serious global issue. Even in a low concentration (from ng/L to low μg/L), these compounds are sufficient to cause harmful (in)direct effects on the aqueous organism and humans [4]. Pharmaceuticals in wastewaters come from hospitals, veterinaries, households, and (pharmaceutical) industries [5].

To that end, tolperisone hydrochloride (TLP) is a piperidine derivative and belongs to the group of muscle relaxant pharmaceuticals. TLP affects spasticity through interaction with upper motor neurons (central-acting muscle relaxants) [6]. It is commercially available under different trademarks (e.g., Biocalm^®^, Muscodol^®^, Mydeton^®^, Mydocalm^®^, Mydoflex^®^, Myolax^®^, Myoxan^®^, and Viveo^®^) and used in various treatments of rheumatological, orthopedic and traumatic neurological disorders [7]. Although the toxicological effects on the environment and living organisms of this product have not been thoroughly studied, according to 1907/2006/ EEC/ Article 31 [8], TLP belongs to water hazard class 3 (German Regulation), which means that it is extremely hazardous for water and should not be allowed to reach the ground water, water course or sewage system, even in small quantities. Moreover, it is dangerous to drinking water if even extremely small quantities leak into the ground [8].

Pharmaceuticals and personal care products are a class of developing micropollutants [9]. Municipal wastewater treatment plants (WWTPs) are designed to control and eliminate different compounds from the aqueous environment, such as particulates, carbonaceous substances, nutrients, and pathogens. However, these WWTPs are not efficient enough in removing the micropollutants, so there is a requirement for more powerful and competent techniques in order to eliminate the emerging pollutants, too [10]. Different approaches were examined in order to remove these compounds, such as ionic exchange, adsorption, different separation techniques, biological treatments, etc. However, the advanced oxidation processes (AOPs) are proved to be the most capable of all alternatives because only these methods may remove up to 100% of various drugs [9]. Homogenous and heterogeneous photolysis and photocatalysis belong to AOPs. Photolytic processes are based on the interaction of artificial or natural light with the target molecule, while heterogenous photocatalytic degradation is based on the application of various semiconductors as photocatalysts (such as TiO_2_, ZnO, etc.) with the key advantage of including operation at ambient conditions, as well as the fact that the catalyst itself is inexpensive, commercially available in various crystalline forms, has particle characteristics and is photochemically stable [11,12].

In addition, nanotechnology is a new and emerging field of technology that deals with nanoscale particles (<100 nm). In pollution, especially for water pollution, nanotechnology for clean water is changing the business landscape in both the developed and the developing world. Nanomaterials have remarkably useful properties for the removal of numerous water pollutants, for example, photocatalytic nature, high surface area, high reliability, high aspect-ratio, electrostatic and/or magnetic properties, compressible with constant surface area, tunable pore volume, short intra-particle diffusion distance, hydrophobic and hydrophilic nature, etc. [13].

Photocatalytic reactions are based on the interaction of the semiconductor (photocatalyst) and light of sufficient energy (certain wavelength) to produce highly reactive oxidizing species which interact with the pollutants. For a successful photocatalysis, it is necessary that the energy of light be greater than the band gap of the applied semiconductor. In the case of TiO_2_, the band gap is 3.2 eV, and in the case of ZnO is 3.3 eV. The absorption of a photon excites an electron to the conduction band and generates a positive hole in the valence band. Afterward, these holes take place in oxidation processes (e.g., oxidize OH^−^ or water at the surface to produce •OH radicals) while the electrons in the conduction band can be rapidly trapped by molecular oxygen adsorbed on the semiconductor surface, which is reduced to form superoxide radical anion ($O_{2}^{•-}$) [14,15].

In the heterogenous photocatalytic degradation, besides the type of catalyst, there are various factors that affect the efficiency of the photocatalytic process, such as type of irradiation, catalyst loadings, and length of irradiation. In the experiments of photocatalytic degradation, mostly two types of irradiations are investigated, which are simulated solar irradiation (SSI) and ultraviolet (UV) light. Since sunlight is naturally available and free, this energy source should be exploited and intensively used for activating photocatalysts in a sustainable manner and, in this way, removing the pollutants from the (waste)water. Even though solar irradiation covers a part of the UV spectrum, because of the high band gap energy of the commonly used semiconductors as photocatalysts, this source needs help to provide sufficient degradation. Thus, UV irradiation is also highly interesting as a high-energy source for eliminating pollutants from the environment. Even though UV has better performance, the lack of its natural source results in higher operating expenses and a lower degree of sustainability [16,17]. Considering the catalyst loading, it is known that the reaction rate increases with increasing the amount of photocatalyst in the reaction system up to a certain value, after which the further increase in the semiconductor concentration leads to reduced or has no effect on degradation efficiency [18]. Another factor that affects photocatalytic degradation is the length of irradiation. Both the reaction rate and the photocatalytic efficiency are related to the length of irradiation. Namely, the efficiency of photocatalytic degradation increases with the increased time of irradiation [19]. As mentioned above, it is extremely important to define optimal parameters for the removal of particular pollutant. For this purpose, chemometrics offers tools that include mathematical/statistical approaches which are able to analyze large datasets and optimize the degradation of TLP and obtain useful information about the interactions between different factors (able to quantify mutual synergistic effects). Common techniques used for this purpose are multivariate analysis, data clustering, and mathematical modeling [20].

Various findings showed that pharmaceuticals may have toxic effects on plants, tolperisone being one of them [21]. Even though very important, studies about the toxic effects of TLP on the germination, photosynthetic capacity, and biomass production of plants seem to be very scarce. Alfred et al. [21] proposed four groups of major structural classes of compounds that are related to specific phenotypes. According to them, TLP belongs to group III (affecting photosynthesis), which is a combination of two unrelated clusters, phenyl-piperidine-oxazoles and benzoyl-piperidine, enriched for photosynthetic inhibitors, while TLP alone belongs to group IIIb. Significant overlap was found between photosynthesis and growth inhibitors. However, the relationship between germination, mass, chloroplast pigments, and different pharmaceuticals has been investigated previously. For instance, Finčur et al. [22] studied the effect of two antibiotics (ciprofloxacin and ceftriaxone) after photodegradation in the presence of TiO_2_, ZnO, and MgO nanoparticles on wheat germination and biomass production. Findings showed that the mixtures of each pollutant that formed intermediates had an effect on the further growth of young plants. Additionally, in the study of Rede et al. [23], there were a decreased germination and plant mass observed, mostly in the early phase of growth. Furthermore, the concentration of photosynthetic pigments was also lower in the plants treated with different active pharmaceutical ingredients (API). That was the main reason for using them to assess their environmental toxicity in these experiments.

The aim of this research was to investigate the environmental toxicity of TLP and formed photodegradation intermediates on the germination, growth of wheat, and concentration of photosynthetic pigments. Likewise, the optimization of the photocatalytic degradation efficiency and its influence on germination was performed. The photocatalytic experiments were carried out in the presence of two types of photocatalysts, namely TiO_2_ Hombikat (TiO_2_) and ZnO, using SSI and UV light. Moreover, LC–ESI–MS analysis was also conducted in order to prove the removal efficiency of TLP, as well as to detect the eventual degradation intermediates after successful photodegradation. The data obtained both from the photocatalytic experiments and the experiments of toxicity on the plant were processed and discussed by applying chemometrics. In order to optimize the photodegradation efficiency and germination time, as well as to discover the possible interactions between different photocatalytic reaction parameters, a full factorial design was applied. Assessments evaluated the percentage of TLP after irradiation (the two most important parameters), germination time, plant mass, and pigment content. Additionally, the following factors were used as a source of variation in the dataset: type of catalyst (TiO_2_ and ZnO), catalyst loading (0.5, 1.0, and 2.0 mg/mL), germination time (24, 48, and 72 h), irradiation time (30, 60, and 120 min), as well as the type of irradiation (SSI and UV).

## 2. Materials and Methods

### 2.1. Materials and Reagents

**Figure 1:**
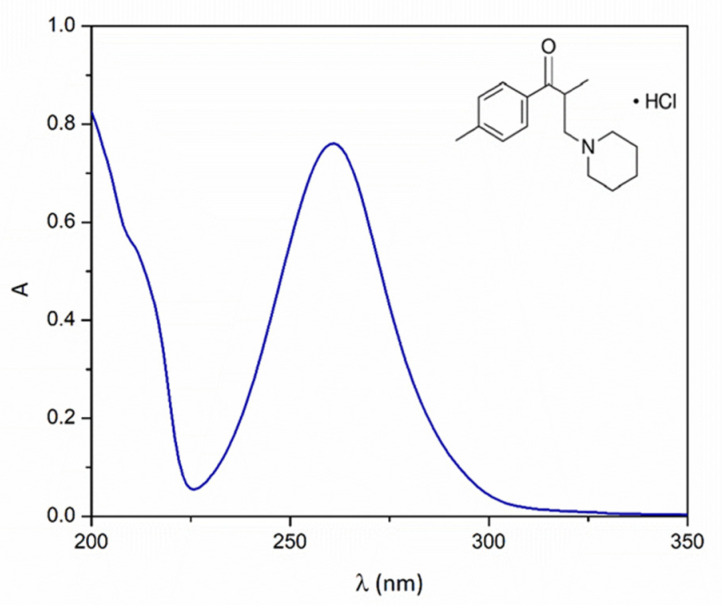
Absorption spectrum and structural formula of TLP.

TLP (CAS No 3644-61-9, C_16_H_24_ClNO, M*r* = 281.82, 99.5% purity, Goodwill Pharma, Subotica, Serbia) was used as the API in the experiments of photocatalytic degradation and toxicity. The absorption spectrum and the structure of this compound are shown in Figure 1. The following chemicals were used as the components of the mobile phase for liquid chromatography: acetonitrile (99.9%, Sigma-Aldrich, St. Louis, MO, USA) and orthophosphoric acid (85%, pro analysis, Sigma–Aldrich, St. Louis, MO, USA).

In LC–ESI–MS, acetonitrile (99.9%, Sigma-Aldrich, St. Louis, MO, USA) and formic acid (≥95%, pro analysis, Sigma–Aldrich, St. Louis, MO, USA) were used as the components of the mobile phase.

The aqueous solution of TLP (0.05 mM) was prepared by dissolving the appropriate mass of this substance in ultrapure water, and the solution was protected from light at ambient temperature.

In the experiments of photocatalytic degradation, TiO_2_ Hombikat (CAS No 13463-67-7, surface area 35–65 m^2^/g and 21 nm primary particle size, anatase, Sigma-Aldrich Chemie GmbH, Steinheim, Germany) and ZnO (CAS No 1314-13-2, ≥99%, Sigma-Aldrich Chemie GmbH, Steinheim, Germany) were used as photocatalysts. Finčur et al. [24] have carried out investigations in order to characterize the above-mentioned ZnO nanoparticles. Based on the scanning electron microscopy images, it can be concluded that the smallest particle has a diameter of approximately 40 nm. Moreover, the Brunauer–Emmett–Teller analysis showed a specific surface area of 6.5 m^2^/g.

### 2.2. Sample Preparation

Photodegradation experiments were performed in photochemical cells made of Pyrex glass (total volume of ca. 40 mL, liquid layer thickness 35 mm), with a plain window on which the light beam was focused. The cell was also equipped with a magnetic stirring bar and water-circulating jackets. The experiments under SSI were carried out using a 50 W halogen lamp (Philips), whereas, in the experiments with UV irradiation, a 125 W high-pressure mercury lamp (Philips, HPL N) was used. The UV energy fluxes were measured using a Delta Ohm HD 2102.2 (Padova, Italy). The radiometer was fitted with the LP 471 UVA sensor (spectral range 315–400 nm), and in the case of the Vis, the energy radiometer was fitted with the LP 471 RAD (spectral range 400–1050 nm). The photon flux for the halogen lamp was 63.85 mW/cm^2^ for visible radiation and 0.22 mW/cm^2^ for the UVA region, while in the case of the mercury lamp, the photon flux was 5.304 mW/cm^2^ for the UVA region.

Experimental conditions were chosen on the basis of our previous results [22,24,25]. Namely, in the past optimal irradiation time for organic pollutants (active ingredients of pesticides and API), removal by using heterogeneous photocatalysis was mostly achieved between 30 and 60 min under UV light, while in the case of SSI, it took a longer time. In addition, optimal TiO_2_/ZnO loading was in the range of 0.5–2.0 mg/mL. Thus, the photocatalytic experiments were carried out using 30 mL of 0.05 mM solution of TLP with 15 mg (γ = 0.5 mg/mL), 30 mg (γ = 1.0 mg/mL), and 60 mg (γ = 2.0 mg/mL) of TiO_2_/ZnO. The suspension was stirred in the dark for 5 min before irradiation to achieve adsorption-desorption equilibrium and uniform particle size of the catalyst. Moreover, the reaction mixture was kept at 25.0 °C using a circular thermostat with a constant stream of O_2_ (3.0 mL/min).

The samples of TLP taken after different times of irradiation (30, 60, and 120 min) using SSI/UV light and different catalyst loadings (0.5 mg/mL, 1.0 mg/mL, and 2.0 mg/mL) were firstly filtrated through Black ribbon filter (Schleicher&Schuell, φ 125 mm) in the case of TiO_2_ and Albet 135 filter (φ 20–25 μm) in the case of ZnO. Afterward, they were also filtrated using Millipore (Millex-GV, Burlington, MA, USA, 0.22 µm) membrane filter to remove all the catalyst particles. The prepared samples were further used in the investigation of the efficiency of photocatalytic degradation and toxicity. In the toxicity investigation, wheat seeds were germinated both in the presence of TLP solution (0.05 mM) and the filtrated suspensions of TLP and formed intermediates after irradiation.

### 2.3. Toxicological Assessments

**Figure 2:**
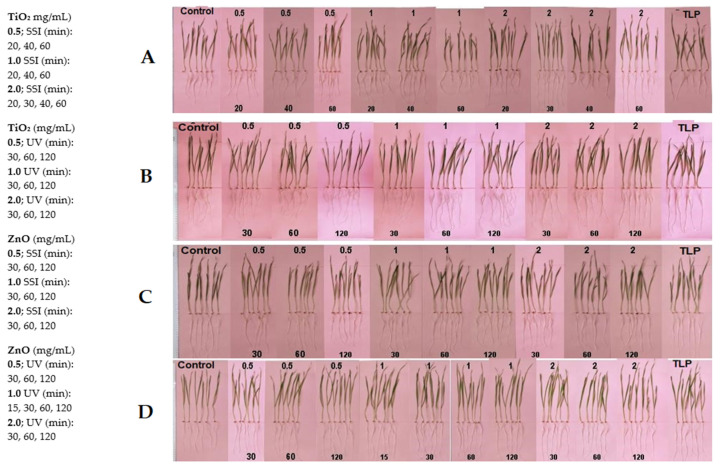
Images of wheat after 8-days treatment with various solutions of TLP under irradiation: TiO_2_ and SSI (**A**); TiO_2_ and UV (**B**); ZnO and SSI (**C**); ZnO and UV (**D**).

In the toxicity experiments, ultrapure water (no TLP) and a solution of TLP (100%) were both used as controls. Such controls were used to distinguish the effects on germination and growth of pure TLP on the one hand and mixture of TLP and intermediates that remained after irradiation on the other. In this way, it was possible to relate the efficiency of photodegradation to toxicity via germination and early growth of wheat. An experiment was performed similarly as explained by Finčur et al. [22]. In the toxicity assessment, the following factors were examined in order to prove the toxicity of TLP and its degradation intermediates: shoot mass (=above-ground mass) per plant (m(AG/P)/[mg]); root mass per plant (m(R/Plant)/[mg]); shoot/root ratio (m(AB/R)/[mg]); germination; concentration of chloroplast pigments. These factors were selected as they give the most relevant image of the health and proper physiological functionality of the plants. Wheat cultivar NS40S was used in the experiments. To assess the effect of TLP and its derivatives on germination, 30 seeds were placed on a filter paper in a Petri dish (R = 9 cm) to which was added 7.5 mL of either ultrapure water, 0.05 mM TLP or solution containing products of its degradation by UV or SSI in the presence of either TiO_2_ or ZnO in three different loadings (0.5 mg/mL; 1.0 mg/mL and 2.0 mg/mL) (Figure 2A–D). To ensure the accurate use of the seeds, every experiment and the planting process were overseen by qualified agronomists from our research team. Petri dishes were prepared in three replications for each treatment, covered with a lid, and placed in the incubator at 26 °C. The percentage of germinated seeds was recorded after 24, 48, and 72 h. Seedlings were then transplanted in plastic pots (V = 750 mL) filled with ½ strength Hoagland solution [26]. Sixteen plants were planted in each pot, and 3 pots were set with seedlings deriving from each treatment. Pots were placed in a growth chamber (RK-340 CH, Kambič), and plants were exposed to 12 h day/night period (light provided by FLUORA 18W/77 lamps), 23 °C/19 °C temperature regime, 45% humidity, and 80% ventilation. After 8 days in the chamber, when plants were 11 days old, their root and shoot FW and concentration of photosynthetic pigments were assessed. In addition, the concentration of MDA (a measure of the integrity of cell membranes), H_2_O_2,_ and free proline were assessed, but since there were no differences in their concentrations, these parameters were belated in further analysis. The concentration of photosynthetic pigments was measured in acetone extracts of freshly harvested leaves using molar extinction coefficients, according to Holm [27] and von Wettstein [28]. Dry weight of roots and shoots was assessed after drying plant material to constant mass at 60 °C.

### 2.4. Analytical Procedures

In order to follow the efficiency of photocatalytic degradation of TLP under different experimental conditions, a high-pressure liquid chromatograph with a diode array detector (UFLC-DAD, Shimadzu Nexera, Tokyo, Japan) (wavelength of TLP maximum absorption at 258 nm) equipped with Inertsil^®^ ODS-4 column (2.1 mm × 50 mm i.d., particle size 2 μm, 40 °C) was used. Prepared samples (20 µL) were injected and analyzed. The mobile phase (flow rate 0.8 mL/min) was a mixture of acetonitrile and water (32:48, *v*/*v*, pH 2.56), while the water was acidified with phosphoric acid so that the mass fraction of phosphoric acid was 0.1%. The absorption spectrum of TLP was analyzed using a T80+ UV/Vis Spectrophotometer, PG Instruments Ltd.

For the reversed-phase liquid chromatography–electrospray ionization mass spectrometry (LC–ESI–MS) evaluation of TLP decay and intermediates, 2 µL samples were analyzed on a Shimadzu LCMS-8040 with electrospray ionization triple-quadrupole MS/MS, using Phenomenex Kinetex C18 column (50 mm × 2.1 mm i.d., particle size 2.6 µm). The column oven temperature was set at 40 °C, the heat block temperature was set at 400 °C, and the temperature of the desorption line was 250 °C. The mobile phase (flow rate 0.2 mL/min) consisted of 0.1% aqueous formic acid (A) and 0.1% formic acid in acetonitrile (B) (50%:50%). Analytes were ionized using an electrospray ion source, a capillary voltage of 4.0 kV, nitrogen as a drying gas (flow 15 L/min), and nebulizer gas. Full scan mode (*m*/*z* range 84–300, scan time 2 min) in positive mode was used to select ions for each sample. Then, the tolperisone standard and each treated sample were analyzed in SIM mode (under the same conditions mentioned above, at *m*/*z* = 284) in order to determine the response of the target molecule, i.e., to quantify its residual amount in treated samples.

The ultrapure water for the preparation of all solutions was provided by Adrona water purification system (LPP Equipment AG, Uster, Switzerland).

### 2.5. Statistical Analysis

Experiments were designed as a full factorial randomized experimental design [29]. Dependent variables were: (i) number of germinated seeds (n = 324); (ii) percentage of TLP after irradiation (n = 324); (iii) shoot mass (=above-ground mass) per plant (n = 144); (iv) root mass per plant (n = 144), (v) shoot/root ratio (n = 144). Independent variables were: (i) type of catalyst (CT; TiO_2_/ZnO); (ii) catalyst loading (CL; 0.5, 1.0, and 2.0 mg/mL); (iii) germination time (GT; 24, 48, and 72 h); (iv) irradiation time (IT; 30, 60, 120 min); and (v) source of light (SLG; SSI and UV light). All samples were analyzed minimally as triplicates.

Descriptive statistics provided information about the dataset. Continuous variables were tested by multivariate analysis of variance (MANOVA). Pearson’s linear correlation tested the relation between the pairs of continuous variables. Linear regression was employed to build and compare mathematical models. The significance levels for all tests were α ≤ 0.05, all variance inflation factors were ≤5, and lack of fit tests was insignificant. Only statistically significant predictors were retained in the models (α ≤ 0.05). Clustering was conducted by application of the Chi-square automatic interaction detection (CHAID) method. Analyses were performed with IBM SPSS Statistics (v.24; IBM Corporation, 1 New Orchard Road, Armonk, New York 10504-1722, United States 914-499-1900). Statgraphics Centurion XVII was used to build and compare mathematical models (Statpoint Technologies, Inc., Warrenton, VA, USA).

## 3. Results and Discussion

### 3.1. Exploratory Clustering of Samples in the Datasets

**Figure 3:**
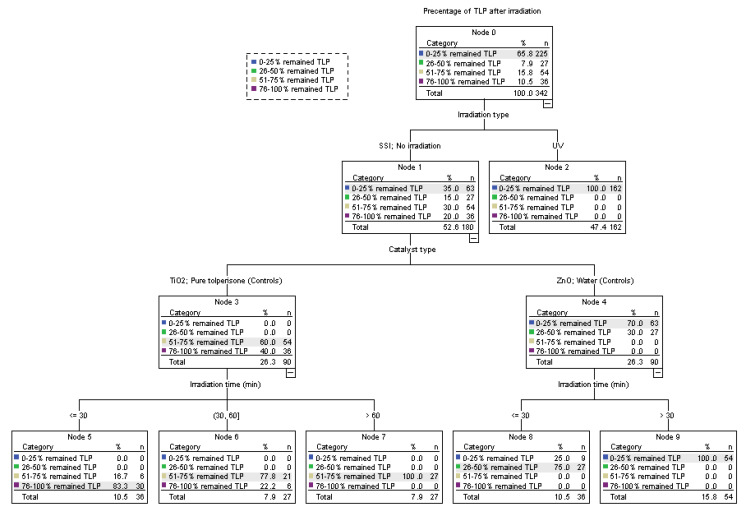
Classification of the samples based on the percentage of tolperisone (TLP) after irradiation.

**Figure 4:**
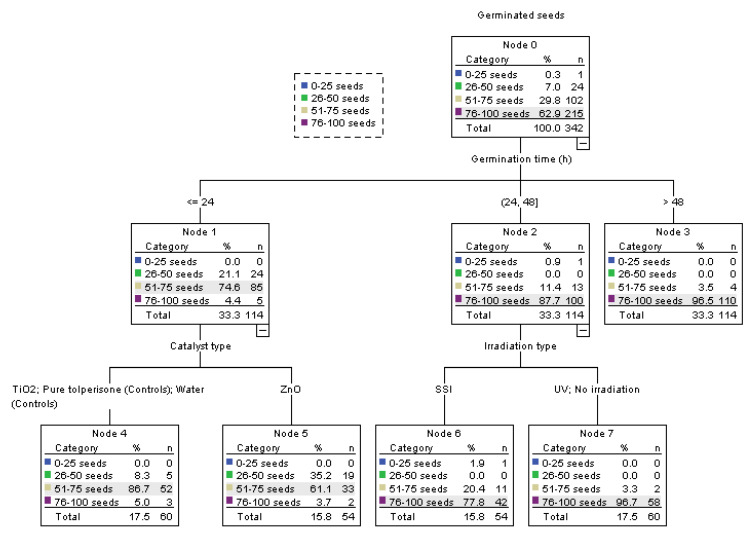
Classification of the samples based on germination.

To obtain the direction of future chemometric analysis and overall picture of the dataset, exploratory classification of the samples was conducted by two most important dependent variables, namely the percentage of TLP after irradiation and the number of germinated seeds. In the former case, samples were clustered to 9 nodes where the right side of the tree contained samples that had between 0–50% of TLP, implying that samples with longer irradiation time, ZnO and UV light will likely have lower values for percentage of TLP after irradiation (Figure 3). As expected, control samples that were germinating in the presence of ultrapure water were clustered here as well. The number of samples that had better germination (7 nodes) seems to be positively associated with UV light, TiO_2_, longer germination, and complete absence of irradiation (associated with both control samples; Figure 4). Control samples with ultrapure water, on average, had higher seed count (82.67 ± 1.00 seeds) than controls with TLP that had 78.89 ± 1.00 seeds (*p* = 0.01). The number of germinated seeds increased with longer germination time for all controls (*p* ≤ 0.01). Further analyses were conducted to evaluate the exact relationships among the rest of the variables.

### 3.2. Photocatalytic Degradation of TLP

**Figure 5:**
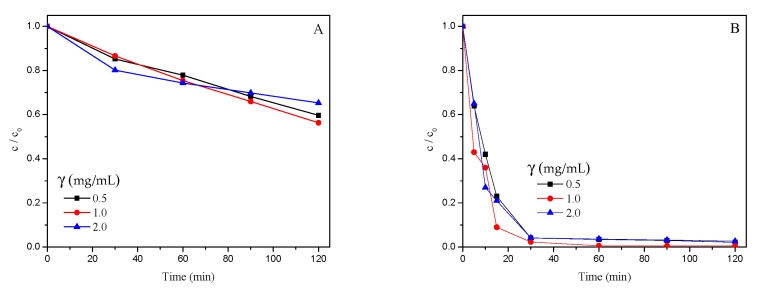
Kinetics of photocatalytic degradation of TLP (0.05 mM) in the presence of TiO_2_ under SSI (**A**) and UV irradiation (**B**).

**Figure 6:**
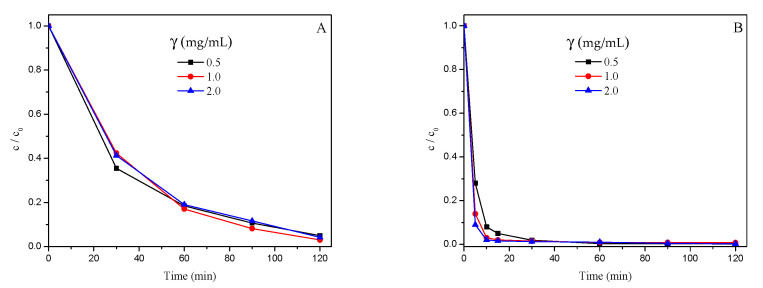
Kinetics of photocatalytic degradation of TLP (0.05 mM) in the presence of ZnO under SSI (**A**) and UV irradiation (**B**).

First of all, the results of photocatalytic studies were processed, and they are shown in Table 1. According to them, it can be seen that, on average, samples with ZnO (11.14 ± 0.12%) had a lower percentage of TLP after irradiation than samples with TiO_2_ (35.07 ± 0.12%; Table 1). The investigated catalysts have similar properties, but in some cases, they act differently [30]. The higher efficiency of ZnO in the photodegradation of TLP can be explained by the fact that even though ZnO (3.3 eV) has similar band gap energy like TiO_2_ (3.2 eV), one of its greatest advantages is the ability to absorb a wider range of solar spectrum and a higher number of light quants [31]. Additionally, ZnO has many specific properties such as good transparency, considerable free-exciton binding energy (60 meV), high electron mobility (200–1000 cm^2^/(V s)), and strong room-temperature luminescence as compared to TiO_2_ nanomaterials. Consequently, the lowest remaining TLP after irradiation was in samples with TiO_2_ (*p* ≤ 0.01) that were irradiated with UV light (0.6 ± 0.1%), while SSI samples had averages that were much higher (69.5 ± 0.1%). Similarly, ZnO had better performance with UV light, where TLP after irradiation remained at 1.5 ± 0.2% and for SSI was 20.8 ± 0.2%. Better efficiency with UV irradiation is the consequence of higher number of photons in the UVA region as compared to the SSI, which results in the formation of more, highly reactive species in the reaction mixture [32]. Additionally, the reaction rate is related to the length of irradiation and the increased irradiation time, as it follows the pseudo-first-order kinetics, decreases the reaction rate because of the additional competition for degradation between the reactant and the intermediate products [19]. The kinetics of photocatalytic degradation of TLP under various experimental conditions is shown in Figure 5A and B (TiO_2_) and Figure 6A and B (ZnO). Furthermore, especially in the case of TiO_2_, it is necessary to use a source of irradiation that provides wavelengths shorter than 380 nm in order to photo-excite this catalyst and secure the electron–hole pair separation, however, even using this kind of irradiation, almost 90% of the formed electron–hole pairs recombine, and the photocatalytic activity gets weaker [33].

Somewhat unexpectedly, for both types of catalysts, with increasing their loading also increased the percentage of TLP after irradiation in the samples. Accordingly, for TiO_2_ at 0.5, 1.0, and 2.0 mg/mL loading, TLP = 33.4 ± 0.2%, 35.4 ± 0.2%, and 36.5 ± 0.2%, respectively (*p* ≤ 0.01). These values were lower for ZnO and equaled 10.4 ± 0.2%, 11.6 ± 0.2%, and 11.4 ± 0.2%, respectively (*p* ≤ 0.01).

The application of UV light vs. SSI tremendously decreased the TLP in the samples (from 1.04 ± 0.12% to 45.16 ± 0.12%, Table 1). Increment of the catalyst loading increased the percentage of TLP in the samples from 21.90 ± 0.15% for 0.5 mg/mL to 23.94 ± 0.15% for 2.0 mg/mL (Table 1). On the other hand, longer irradiation time decreased the percentage of TLP from 30.72 ± 0.15% for 30 min down to 15.71 ± 0.15% at 120 min (Table 1), and this was observed for both of the photocatalysts. Generally, if the concentration of the catalyst is higher, there is an increased number of active sites on the catalyst’s surface for the photocatalytic reactions, and this happens until then all the particles are fully illuminated, which raises the reactive species in the solution (e.g., hydroxyl and superoxide radicals). On the other hand, above the optimal concentration, a screening effect of excess particles occurs. Hence, a part of the photosensitive surface will not be available for photocatalytic processes and consequently hindering or even reflecting the penetrating light [18]. Moreover, if the loading is beyond the optimum, there is a possibility of agglomeration of catalyst particles; hence, the part of the catalyst surface becomes unavailable for photon absorption, and the degradation rate decreases [30,34]. In the case of higher loadings, there is an increased number of available active sites on the catalyst’s surface. These processes could occur in our experiments with increasing the catalyst loading also increased the percentage of TLP after irradiation for both types of catalysts.

**Figure 7:**
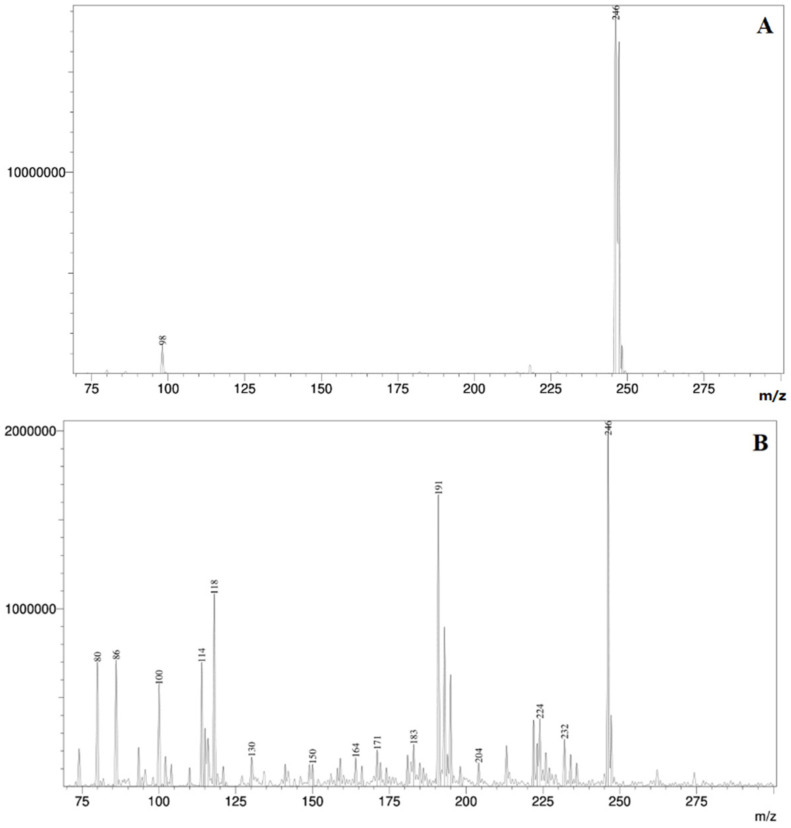
LC–MS spectrum of TLP standard (**A**); sample in the presence of catalyst (1.0 mg/mL) after 60 min of irradiation under UV irradiation (**B**).

In order to prove the removal of TLP and detect the eventual photodegradation intermediates, LC–ESI–MS analysis was performed. Firstly, in accordance with the statistical analysis (Section 3.6), the LC–MS results proved the successful removal of TLP from the aqueous suspension (Figure 7). Namely, after 60 min of UV irradiation with TiO_2_ loading of 1.0 mg/mL, 96.45% of TLP was degraded. Furthermore, the LC–MS spectrum also confirmed the presence of different intermediates in the samples after the irradiation, which additionally proves that the photocatalytic removal was efficient, and TLP was degraded into smaller components (Figure 7). In our future research, we would like to completely investigate and identify all the formed intermediates and to further examine their effect on living organisms.

### 3.3. Germination

Plant germination is the first essential step in plant development. This initial step, followed by uniform sprouting, is very important for the successful crop growth further during the vegetative season. Germination starts with the imbibition of a seed, which is essential for the activation of the enzymes and plant hormones involved in reactions, thus leading to the growth of an embryo and, finally, the protrusion of ridicule through the testa [35]. Therefore, if the water which is in contact with the seed during imbibition contains dissolved substances, they may intervene with the uptake of water (e.g., salts, acting as osmotic [36]) and/or they may enter into the seed and affect its germination and/or impact further plant growth. These findings are sometimes also successfully used in agricultural production, i.e., in the process of seed priming, which may have stimulating effects which enhance plant growth [37,38,39]. Therefore, that is why we analyzed the impact of TLP and intermediates formed during the degradation under different lights and catalysts on the germination of wheat.

**Figure 8:**
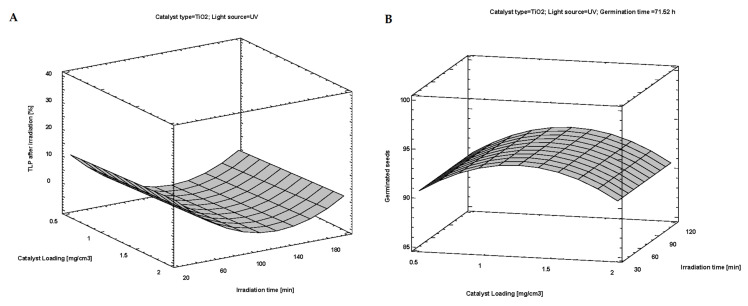
Optimal degradation of TLP after irradiation (**A**); optimal parameters for germination of wheat seeds (**B**).

Accordingly, from Table 1, it can be seen that experiments that used TiO_2_ (79.37 ± 0.64) had a higher number of germinated seeds than ZnO (76.85 ± 0.64). There was no difference in the number of germinated seeds among SSI (77.55 ± 0.64) and UV light (78.67 ± 0.64). Meaning both photocatalysts will work fine under SSI and UV light. An increase in the photocatalyst loadings made sense until 1.0 mg/mL as it increased the number of germinated seeds, while after that point (e.g., at 2.0 mg/mL), additional increase in loadings started to revert to the initial number of seeds at 0.5 mg/mL or just remained constant and this was true for both photocatalysts (Table 1). According to Figure 8A, it can be seen that the optimal catalyst loading in the photocatalytic experiments was 1.0 mg/mL. Taking this into account, the increased number of germinated seeds under these conditions could be explained by the successful removal of TLP and the photodegraded intermediate(s), whereas 0.5 mg/mL and 2.0 mg/mL catalyst loadings were not. The successful degradation of TLP under optimal conditions was additionally proved by the LC–MS spectrum (Figure 7). In the end, this led to a decreased or constant number of germinated seeds. According to these findings, it can be concluded that TLP had an effect on wheat germination because, in the experiments where the composition of imbibition solution was not optimal, the number of germinated seeds was decreased.

For germination, irradiation time had no influence, and already at 30 min of irradiation average of 77.25 ± 0.78 remained constant through 60 and 120 min of irradiation, regardless of its type (Table 1). The possible reason for this is perhaps that in the first 30 min of irradiation sufficient amount of TLP was removed to decrease toxicity. Additionally, it is possible that formed intermediates were not as toxic when compared to the initial compound, so the toxicity was weaker and had no significant influence on germination (Table 1). Indeed, the average TLP remaining in samples after 30 min of any irradiation was 30.72 ± 0.15 (i.e., 70% was degraded).

Germination time was positively associated with the number of germinated seeds, and the longest germination time of 72 h yielded the highest number of germinated seeds (88.96 ± 0.78). Under natural conditions, the germination time could have various influences on the number of germinated seeds. Namely, plants in nature with longer germination times can be subjected to different, changeable biotic and abiotic factors such as photoperiod, temperature, substrate moisture, soil nutrient level, and competition [40]. In our case, these factors were controlled, and the positive effect on the growth with increasing the time of germination showed that TLP was eliminated successfully, and the laboratory conditions were appropriate for the growth of wheat, too. ZnO photocatalyst had constant number of germinated seeds for both types of light sources, while TiO_2_ performed better under UV light (81.73 ± 0.8; *p* ≤ 0.01) and worse with SSI (77.57 ± 0.80; *p* ≤ 0.01), than the controls. These findings are in accordance with the results of the photocatalytic studies (Section 3.2), where the higher efficiency of UV irradiation than SSI in the degradation of TLP and possible intermediates was discussed in detail. Both photocatalysts had the same effect on germination regardless of the length of irradiation (78.11 ± 0.93; *p* = 0.31). Additionally, both of the semiconductors, on average, removed +65% of TLP from the samples (Table 1). This might be sufficient to negate the physiological toxicity of wheat plants or at least alleviate toxicity at the germination stage of wheat. Considering only this parameter, this strongly implies that photocatalytic degradation is a suitable approach to reduce the toxicity of TLP in environmental and waste waters [41,42,43].

### 3.4. Parameters of Plant Mass

Seedlings deriving from seeds that germinated in the presence of TLP and its photodegradation intermediates were further grown by the method of water cultures to assess possible impacts imposed during germination on the further growth of young wheat plants.

Here, catalyst type had no influence on the shoot fresh weight (m(AG/P)/[mg]) (Table 2). However, seedlings deriving from seeds exposed to TLP treated by UV light had higher shoot mass (m(AG/P) = 245.33 ± 1.92 mg) than SSI (m(AG/P) = 221.71 ± 1.92 mg). An increase in the photocatalyst loadings made sense until 1.0 mg/mL, as after that point (e.g., at 2.0 mg/mL) with an additional increase in loadings m(AG/P) = 236.88 ± 2.35 mg, and remained constant as with 1.0 mg/mL. These results are in line with those obtained for germination and can be interpreted identically while implying an emerging pattern in the dataset.

Accordingly, considering that the optimal catalyst loading was 1.0 mg/mL (see Section 3.6), it is obvious that under these conditions, the 100% removal efficiency of TLP and probably its photodegradation intermediates was reached. Hence, there was the weakest toxic effect observed, which positively affected the plant mass. The lowest shoot fresh weight (m(AG/P) = 224.21 ± 2.35 mg) was with 60 min of irradiation while extending the length of irradiation (to 120 min) yielded a surge of m(AG/P) = 238.56 ± 2.35 mg similar to 30 min of irradiation (Table 2). Interestingly, m(AG/P)/[mg] was not the same for both types of light sources over different times of irradiation. While the time of irradiation had no influence on m(AG/P)/[mg] for UV light (245.32 ± 2.90 mg; *p* = 0.15), for SSI, it dropped between 30 (229.58 ± 3.57 mg; *p* ≤ 0.01) and 60 min (207.38 ± 3.57 mg; *p* ≤ 0.01) only to revert to initial value at 120 min (228.17 ± 3.57 mg; *p* ≤ 0.01). Further elongation of irradiation beyond 60 min reversed this pattern, likely due to further addition of electromagnetic energy and assuring electrostatic changes of the active site (further release of electrons) towards increased affinity for reactant molecules (e.g., TLP). The result is fostering photocatalysis of TLP and reversing m(AG/P) (i.e., toxicity) to its initial value. According to the differences between SSI and UV irradiation (Section 3.2), it is expected that under SSI, it may take more time to remove TLP/intermediates, whereas degradation might be less of a linear process [44].

For SSI to observe the change in the m(AG/P)/[mg], it was not sufficient to increase catalyst loading from 0.5 mg/mL (213.54 ± 3.57 mg) to 1.0 mg/mL (222.17 ± 3.57 mg) where m(AG/P)/[mg] remained constant (*p* = 0.09). However, quadruplication of the initial value for factor loading showed a significant difference for m(AG/P)/[mg] = 229.43 ± 3.57 mg; *p* ≤ 0.01) from initial loading. Since the sunlight is readily available (e.g., average annual sunshine, similar to average EU sunshine, in Belgrade is 2112 h [45]), it is obvious that the natural photodegradation pathways of the pollutants in the environment should be based on SSI. Unfortunately, because of the photocatalysts’ drawbacks mentioned in Section 3.2, we cannot effectively use sunlight. Taking this into account, the higher m(AG/P) at 2.0 mg/mL under SSI can be explained by the fact that SSI is a weaker source of radiation than UV, and it may require a higher amount of catalyst to reach the same/similar results as in the case of the UV irradiation [17]. For UV pattern was somewhat clearer (*p* ≤ 0.01), where doubling of the initial value yielded an increase in m(AG/P)/[mg] from 229.50 ± 2.90 mg to 251.58 ± 2.90 mg (*p* ≤ 0.01), only to remain constant at factor loading at 2.0 mg/mL with m(AG/P) = 252.92 ± 2.90 mg (*p* = 0.42). Connecting with the above-mentioned, the UV irradiation is stronger and more efficient, so the higher m(AG/P) will be already reached at smaller catalyst loading [19].

Furthermore, root fresh weight per plant (m(R/Plant)) was higher for TiO_2_ than for ZnO (101.58 ± 1.69 mg and 93.36 ± 1.69 mg, respectively), which represented a smaller increase (9%; Table 2). Plants deriving from seeds germinated in the presence of UV–irradiated-TLP had higher root fresh weight than in the presence of SSI-light (m(R/Plant) = 101.83 ± 1.69 mg and 93.11 ± 1.69 mg, respectively; Table 2). Regardless of the light source used, m(R/Plant) remained constant for TiO_2_ (101.58 ± 2.45; *p* = 0.67), while for ZnO m(R/Plant) = 102.83 ± 2.35 mg it was higher for UV than for SSI (83.89 ± 2.35 mg; *p* ≤ 0.01). Taking into account the earlier statements (Section 3.2), it is obvious that the applied photocatalysts had weaker activity in the solar spectrum (SSI), so the lower toxicity and higher m(R/Plant) are attained under UV irradiation.

The highest m(R/Plant) = 102.00 ± 2.07 mg was recorded at 1.0 mg/mL, and after that point (e.g., at 2.0 mg/mL), remained constant with an additional increase in loadings (106.69 ± 2.07 mg; Table 2). Interestingly, the highest m(R/Plant) = 98.31 ± 2.07 mg was with 30 min of irradiation, while extending the length of irradiation (to 60 min) yielded a drop to 91.40 ± 2.07 mg (Table 2). Further extension of irradiation time yielded an increase in m(R/Plant) = 102.71 ± 2.07 mg, which was not statistically different from m(R/Plant) at 30 min. A pattern similar to this was observed earlier with m(AG/P) and could be explained in a similar manner. The decreased yield during the irradiation was perhaps the result of the incomplete degradation of the mixture of TLP and probably more toxic intermediates, which was reached after 120 min of irradiation and resulted in an increased m(R/Plant). For different catalyst loadings, different times of irradiation did not modify the average of m(R/Plant) = 97.47 ± 3.61 (*p* = 0.38).

Since a variety of factors may influence different shoot and root growth, their ratios were calculated and analyzed. To that end, m(AB/R) = 2.57 ± 0.03 mg was higher for ZnO than for the TiO_2_ where m(AB/R) = 2.38 ± 0.03 mg (Table 2). One such difference is the fact the treatment with ZnO had a higher toxic effect on the root than on the shoot of wheat.

Irradiation type had no influence on m(AB/R) (Table 2). Opposite to germination, m(AG/P) and m(R/Plant), an increase in the catalyst loadings caused a drop of m(AB/R) parameter, up until 1.0 mg/mL where it was equal to 2.39 ± 0.04 mg, and after that point (e.g., at 2.0 mg/mL), additional increase in loadings stabilized this drop (m(AB/R) = 2.33 ± 0.04 mg) to value of 1.0 mg/mL (Table 2). This can be explained by the increase in the catalyst loading the degradation efficiency of TLP increases up to an optimum value; however, a further increase has no effect since all the available light has already been used [46]. Elongating irradiation time had no difference on m(AB/R) (Table 2). Different types of photocatalyst under different loadings did not modify m(AB/R) (2.48 ± 0.06 mg; *p* = 0.22).

### 3.5. Concentration of Photosynthetic Pigments

Pigmentation was analyzed to assess whether the treatments applied only during germination affected the subsequent growth of plants. In other words, plant growth relays heavily on photosynthetic apparatus, whose constituents are photosynthetic pigments; hence we measured concentrations of Chlorophyll a (Chl a), Chlorophyll b (Chl b), and carotenoids (car) to gauge the toxicity of TLP. This made sense as carotenoids, besides having the role in broadening the specter of sunlight that can be used in photosynthesis, protect chlorophylls from photooxidation, and thus act also as antioxidants [47,48,49,50]. Oxidants and antioxidants are known to be triggered by different kinds of stressors, and therefore we measured concentrations of MDA, H_2_O_2_, and free proline, which indicated the presence of stress-induced reactions in plant tissues. However, since differences were recorded only in concentrations of photosynthetic pigments and biomass, evaluation of other parameters was not reported. Since a variety of factors may influence different shoot and root growth, their ratios were calculated as well.

The applied catalysts affected concentrations of photosynthetic pigments. Treatments of TLP by ZnO resulted in plants containing higher concentrations of chlorophylls in comparison to treatments with TiO_2_ (Table 2). A similar effect had UV light in comparison to SSI. In more detail, it was found that the content of Chl a with ZnO (6.06 ± 0.10) was higher than in the case of TiO_2_ (5.74 ± 0.10; Table 2). Furthermore, this content was higher under UV light (6.56 ± 0.10) than under SSI light (5.25 ± 0.10; Table 2). Because of the higher photocatalytic activity of ZnO (Section 3.2), it is very likely that TLP toxicity was lower than with TiO_2_ (Section 3.2), which in turn, increased synthesis of photosynthetic pigments implied healthier plant with higher potential for growth. However, pathways underlying this mechanism in wheat are not readily available in the literature.

Similar to m(AG/P)/[mg] and m(R/Plant)/[mg], Chl a at 0.5 mg/mL of catalyst loadings (5.69 ± 0.13) increased with an increase to 1.0 mg/mL of loadings (6.18 ± 0.13) where it was the highest, but only to drop to the initial value at 2.0 mg/mL of catalyst loadings (5.83 ± 0.13) or that of 0.5 mg/mL of loadings (Table 2). These findings were in accordance with the optimal conditions of the photodegradation process (Figure 8A). 

For different types of catalysts, different catalyst loadings did not modify Chl a (5.90 ± 0.15; *p* = 0.19). For both types of catalyst (for ZnO = 6.26 ± 0.11, *p* ≤ 0.01; TiO_2_ = 6.86 ± 0.14, *p* = 0.02), UV light had higher values for the Chl a than SSI (for ZnO = 5.87 ± 0.11, *p* ≤ 0.01; TiO_2_ = 4.62 ± 0.14, *p* = 0.02). For SSI light source, Chl a remained constant regardless of the change in catalyst loadings (5.25 ± 0.19; *p* = 0.1). However, for the UV light, this was not true; instead, Chl a with SSI at 0.5 mg/mL was 6.43 ± 0.10 and peaked at 6.82 ± 0.10 with 1.0 mg/mL and then dropped to a value no different than the initial value (6.42 ± 0.10) at highest loading of 2.0 mg/mL (*p* ≤ 0.01). There was no synergistic influence of the type of catalyst, type of light, and catalyst loading on Chl a (*p* = 0.08). Chl b with ZnO was higher (2.80 ± 0.02) than with TiO_2_ (2.48 ± 0.02, Table 2). The differences in the characteristics of TiO_2_ and ZnO, as well as their pros and drawbacks, were already mentioned in Section 3.2, and according to them, the higher concentration of Chl b is concordant with the greater activity of ZnO. Chl b was not influenced by the type of the light source nor by altering catalyst loadings (Table 2). On the other hand, elongating irradiation time increased the Chl b from what was originally at 30 min (2.65 ± 0.03) to 2.73 ± 0.03 (at 60 min) and finished with final drop below the initial value of 2.54 ± 0.03 at 120 min (Table 2). During the irradiation of TLP suspension, the formation of different intermediates is highly possible. Applying LC–ESI–MS analysis on the samples irradiated under optimal conditions, the presence of photodegradation intermediates was confirmed (Figure 7). Hence, their effect on Chl b is obvious. However, these compounds need to be identified and further investigated, which is going to be the subject of our following research. Plants can, under certain forms of stress, convert Chl b to Chl a (as their structures are very similar), resulting in an increase in the concentration of Chl a [51,52]. These compounds might be more harmful than the initial substance itself, as mentioned.

An interesting pattern was observed for this interaction as type of light source did not modify the Chl b with ZnO (2.80 ± 0.04; *p* = 0.23); however, this was not true for TiO_2_ where higher Chl b was found for SSI (2.53 ± 0.03, *p* ≤ 0.01) than for the UV-light source (2.44 ± 0.03, *p* ≤ 0.01). An interesting explanation for the higher concentration of pigments under SSI than UV. Similar to the damage that UV irradiation does to human tissues, those are happening in plant tissues [53]. To that end, UV irradiation on chlorophyll and photosynthesis was already investigated in earlier studies by Warner and Caldwell [54], who investigated the influence of photon flux density by UV-B (280–320 nm) on the inhibition of photosynthesis. Their findings showed that in the case of UV irradiation, there was a greater depression of photosynthesis observed, while, in the case of solar (visible) irradiation, higher Chl a/b ratios were detected. In another study by authors Zvezdanović et al. [55], the effect of UV irradiation was also investigated on the chlorophyll bleaching. Their conclusion was similar to Warner and Caldwell [54]. Namely, UV irradiation caused irreversible breakdown of chlorophyll, and the bleaching of these pigments was observed. These findings and the fact that TiO_2_ has weaker photocatalytic activity than ZnO could be the reasons for higher content of Chl b under SSI than for the UV-light source. More so, the Chl a:Chl b ratio is a reliable indicator of stress in plants. This ratio is the most stable parameter, irrespective of the stress factor plants are exposed to [56]. Nevertheless, different stress factors may either impair the synthesis of photosynthetic pigments or accelerate their degradation. During the process of Chl degradation, Chl b may be converted into Chl a, thus resulting in the increased content of Chl a [51,52]. In our experiment, plants were under optimal light and nutrient regime, factors that can alter concentrations of photosynthetic pigments. Therefore, changes in their concentrations and ratios can be ascribed to changes in plant metabolism provoked by germination in the presence of applied substances.

The overall concentration of chlorophylls (Chl a+b) with ZnO was higher than with TiO_2_ (7.90 ± 0.13) and 7.33 ± 0.13, respectively) (Table 2) and it was higher with UV light than with SSI (8.29 ± 0.13 and 6.93 ± 0.13, respectively) (Table 2). These results can be explained by the greater photocatalytic activity of ZnO than TiO_2_ and the higher number of photons in UV irradiation, which are responsible for the formation of reactive species for the oxidation of pollutants in the reaction mixtures. Similar to before, the content of Chl a+b at 0.5 mg/mL of catalyst loadings (7.34 ± 0.16) increased with 1.0 mg/mL (8.00 ± 0.16) where it was the highest, only to drop in value with 2.0 mg/mL (7.50 ± 0.16) that was no different from 0.5 mg/mL of loadings (Table 2). The findings are in accordance with Figure 8A, where the optimal conditions of photocatalytic degradation are presented. The highest Chl a+b was at 60 min of irradiation (8.13 ± 0.16) while extending the irradiation to 120 min yielded reversion to initial value from initial value of 7.24 ± 0.16, no different than with 30 min of irradiation (7.47 ± 0.16, Table 2). One other interesting relation was observed for Chl a+b, where type of light source did not modify this value for ZnO (7.90 ± 0.15, *p* = 0.12). However, similar to Chl b, Chl a+b was higher for the UV light (8.52 ± 0.16) than for the SSI, where it equaled 6.13 ± 0.16 at *p* ≤ 0.01. Time of irradiation did not modify the concentration of Chl a+b for different catalyst loadings (7.61 ± 0.24, *p* = 0.10).

Catalyst loading did not modify the content of Chl a+b with TiO_2_ for both types of light sources. However, for ZnO content of Chl a+b under SSI light increased from initial value of 7.27 ± 0.32 with 0.5 mg/mL of loading to 8.59 ± 0.32 with 1.0 mg/mL and then decreased to initial value of 7.34 ± 0.32 with 2.0 mg/mL (*p* = 0.02). The optimal catalyst loading is a very important factor in heterogeneous photocatalytic degradation, as mentioned. According to the optimal photocatalytic conditions (Figure 8A), it is expected that under or above the optimal loading (1.0 mg/mL), there will be smaller amount of TLP and photodegradation intermediates removed, and they can affect the health of plant, which is proved by the decreased number of Chl a+b. Similarly, the content of Chl a+b with ZnO under UV increased from initial value of 7.76 ± 0.17 at 0.5 mg/mL of loading to 8.43 ± 0.17 at 1.0 mg/mL and then decreased to initial value of 8.00 ± 0.17 with 2.0 mg/mL of loading (*p* = 0.04). The same conclusions could be taken in this situation. Both catalysts showed no change for Chl a+b value with changing irradiation time under the UV light, and this was also true for samples with ZnO under SSI light. However, TiO_2_ in combination with SSI light showed significant differences for Chl a+b with different irradiation times (*p* ≤ 0.01). Here value Chl a+b peaked after 60 min of irradiation (7.92 ± 0.35) from what was initially detected at 30 min (5.16 ± 0.35), then it dropped to the initial value after 120 min of irradiation (4.86 ± 0.35). These findings are in accordance with the weaker photocatalytic activity of TiO_2_ under SSI caused by the wide band gap (Section 3.2).

The type of catalyst had no influence on carotenoids, while the car under UV light was higher (1.71 ± 0.03) than with SSI light (1.35 ± 0.03, Table 2). The higher concentration of car under UV is probably the reason for higher removal efficiency of TLP and formed intermediates than with SSI. Similar to before, the car at 0.5 mg/mL of the loading (1.43 ± 0.04) peaked with an increase to 1.0 mg/mL of loading (1.64 ± 0.04), only to drop to a value that was no different than the initial value with further increases to 2.0 mg/mL (1.52 ± 0.04, Table 2). Considering the findings shown in Figure 8A, the results can be explained by the optimal conditions for the photocatalytic experiments. The same as with chlorophylls, the highest car was at 60 min of irradiation (1.62 ± 0.04), while further extension of irradiation to 120 min yielded a drop of car to 1.48 ± 0.04, no different to that of 30 min (1.49 ± 0.03, Table 2). For both types of light sources, catalyst loading did not modify the content of carotenoids in the samples (*p* = 1.00). Decreased number of cars under different times of irradiation probably happened because of the negative effect of the present compound on the pigments in wheat. The TLP and its photodegradation intermediates (Figure 7) could block the proper synthesis of chl and car, which led to a decreased number of pigments.

Ratio Chl a+b/car for ZnO was higher (5.30 ± 0.05) than for TiO_2_ (5.01 ± 0.05, Table 2), which can be caused by the higher efficiency of ZnO semiconductor as a photocatalyst. Similarly, SSI was higher (5.27 ± 0.05) than for UV light (5.04 ± 0.05, Table 2), while catalyst loading and irradiation time had no influence on Chl a+b/car (Table 2). When variations of Chl a+b/car ratios were observed for different catalysts that were irradiated with different light sources, this revealed very interesting (opposite) relations. Hence, when samples with TiO_2_ were irradiated with SSI, they had a higher Chl a+b/car ratio (5.72 ± 0.06) than under UV light (4.29 ± 0.06). For ZnO this was inverse, and for samples irradiated with UV light ratio was higher (5.79 ± 0.08) than for the SSI (4.81 ± 0.08) at *p* ≤ 0.01.

### 3.6. Optimal Parameters for Photocatalytic Degradation of TLP and Germination of Seeds

To find optimal conditions for photocatalytic degradation of TLP and germination of seeds, two mathematical models were created. Predictors for germination of seeds (Seed) and percentage of TLP after irradiation (TLP) were: (i) type of catalyst (CT); (ii) catalyst loading (CL); (iii) germination time (GT); (iv) irradiation time (IT); and (v) source of light (SLG).

Firstly, using an adequate model, the degradation of TLP was predicted and calculated by a linear regression model:

TLP [%] = 39.99 − 11.96 × TiO_2_ − 22.0622 × SSI + 1.23 × CL [mg/mL] − 0.40 × IT [min] + 12.39 × TiO_2_ × SSI + 0.002 × (IT [min])^2^ (1)

Here after adjustment (for a number of predictors), the model predicted 94.61% of the variance while this was similar to R^2^ prior adjustment (94.71%) and a good indication that number of predictors had minimal impact on the prediction of variance. It was calculated from the model that complete degradation of TLP (0%) can be achieved by using TiO_2_ with UV light and loading of 1.0 mg/mL with a minimum length of irradiation of 60.83 min (Figure 8A).

The germination model explained 64.46% of the variance (R^2^-adjusted for d.f.) while R^2^ prior adjustment equaled 65.01% showing that number of predictors had negligible influence on the prediction of variance. Calculated linear regression model was:

Seed = 10.29 − 1.26 × TiO_2_ + 13.82 × CL [mg/mL] + 2.24 × GT [h] − 1.24 × TiO_2_ × SSI − 5.53 × (CL [mg/mL])^2^ − 0.02 × (GT [h])^2^ (2)

Accordingly, the highest germination of 93.41 seeds can be achieved by using TiO_2_ with UV light with catalyst loading of 1.0 mg/mL and germination time of 71.52 h, and minimal length of irradiation of 30 min (Figure 8B).

## 4. Conclusions and Outlooks

In this research, we first determined the optimal conditions for the complete photodegradation of TLP. The findings showed that complete TLP removal can be reached using TiO_2_ as a photocatalyst (1.0 mg/mL) after 60.83 min of UV irradiation. LC–MS results also confirmed the successful removal of TLP, as well as the formation of photodegradation intermediates. However, these results have to be further examined, which is going to be the topic of our next study. Additionally, as expected, we proved that the germination time had no influence on the percentage of TLP after irradiation. Our results also suggested that germination time was not related to the percentage of remaining TLP after irradiation. It is important, as germination is one of the most essential processes in plant development, and according to these findings, TLP had no negative effect in this early phase of plant growth. Similarly, a relation with the percentage of TLP after irradiation was not observed in the cases of m(AG/P), m(R/Plant), m(AB/R), and Chl b. On the other hand, a strongly negative effect of the TLP after irradiation was observed in the case of Chl a Chl a+b, and car, while it was moderately (positively) related with Chl a+b/car. Summarizing these data, we successfully proved the toxic effect of TLP and its photodegradation intermediates on the physiology of wheat. Considering the above-mentioned parameters, this strongly implies that photocatalytic degradation is a suitable approach to reduce the toxicity of TLP in the environment and wastewaters. Based on the reported findings, there are many opportunities in the future that can be the topic of further investigation. We suggested a sustainable method for water purification; however, we used TiO_2_ and ZnO as photocatalysts, which are mostly active only under UV irradiation, as well as TiO_2_ is not fully safe for the environment. Thus, various nanoparticles and special nanomaterials with less toxic effects and higher photocatalytic activity under natural sunlight could be synthesized by green approaches and investigated in the photodegradation of TLP. In addition, the photodegradation pathways of TLP should also be examined in detail to obtain more information about all the possible degradation products and their effects on the environment. Furthermore, since the toxicity of TLP has not been examined, further research should be conducted in order to determine the possible harmful effect of TLP on other organisms too.

## Figures and Tables

**Table 1:** Main influences of independent variables on percentage of TLP after irradiation and wheat germination.

Independent Variables	n	TLP [%]	GS
**Catalyst type**		*p* ≤ 0.01 ^†^	*p* ≤ 0.01 ^†^
TiO_2_	162	35.07 ± 0.12 ^a^	79.37 ± 0.64 ^a^
ZnO	162	11.14 ± 0.12 ^b^	76.85 ± 0.64 ^b^
**Light source**		*p* ≤ 0.01 ^†^	*p* = 0.23 ^‡^
SSI	162	45.16 ± 0.12 ^a^	77.55 ± 0.64 ^a^
UV	162	1.04 ± 0.12 ^b^	78.67 ± 0.64 ^a^
**Catalyst loading [mg/mL]**		*p* ≤ 0.01 ^†^	*p* ≤ 0.01 ^†^
0.5	108	21.90 ± 0.15 ^c^	76.25 ± 0.78 ^a^
1.0	108	23.47 ± 0.15 ^b^	79.95 ± 0.78 ^a,b^
2.0	108	23.94 ± 0.15 ^a^	78.13 ± 0.78 ^a^
**Irradiation time [min]**		*p* ≤ 0.01 ^†^	*p* = 0.43 ^‡^
30	108	30.72 ± 0.15 ^a^	77.25 ± 0.78 ^a^
60	108	22.87 ± 0.15 ^b^	78.66 ± 0.78 ^a^
120	108	15.71 ± 0.15 ^c^	78.43 ± 0.78 ^a^
**Germination time [h]**		*p* = 1.00 ^‡^	*p* ≤ 0.01 ^†^
24	108	23.10 ± 0.15 ^a^	60.65 ± 0.78 ^c^
48	108	23.10 ± 0.15 ^a^	84.72 ± 0.78 ^b^
72	108	23.10 ± 0.15 ^a^	88.96 ± 0.78 ^a^
**Sample mean**	324		

Results are expressed as mean ± standard error. Values represented with different letters are statistically different at *p* ≤ 0.05; ^†^ significant factor in multifactor analysis; ^‡^ not significant factor in multifactor analysis. TLP—percentage of tolperisone after irradiation; SSI—simulated solar irradiation; GS—number of germinated seeds.

**Table 2:** Main influences of independent variables on mass ratios and photosynthetic pigments in wheat.

		Plant Mass (mg Plant^−1^)			Concentration of Photosynthetic Pigments (mg g^−1^ FW) and Their Ratios				
Independent Variables	n	m (AG/P)	m (R/Plant)	m (AB/R)	Chl a	Chl b	Chl a+b	Car	Chl a+b/car
**Catalyst type**		*p* = 0.27 ^‡^	*p* ≤ 0.01 ^†^	*p* ≤ 0.01 ^†^	*p* ≤ 0.01 ^†^	*p* ≤ 0.01 ^†^	*p* ≤ 0.01 ^†^	*p* = 0.53 ^‡^	*p* ≤ 0.01 ^†^
TiO_2_	72	235.00 ± 1.92 ^a^	101.58 ± 1.69 ^a^	2.38 ± 0.03 ^b^	5.74 ± 0.10 ^b^	2.48 ± 0.02 ^b^	7.33 ± 0.13 ^b^	1.54 ± 0.03 ^a^	5.01 ± 0.05 ^b^
ZnO	72	232.04 ± 1.92 ^a^	93.36 ± 1.69 ^b^	2.57 ± 0.03 ^a^	6.06 ± 0.10 ^a^	2.80 ± 0.02 ^a^	7.90 ± 0.13 ^a^	1.52 ± 0.03 ^a^	5.30 ± 0.05 ^a^
**Light source**		*p* ≤ 0.01 ^†^	*p* ≤ 0.01 ^†^	*p* = 0.93 ^‡^	*p* ≤ 0.01 ^†^	*p* = 0.64 ^‡^	*p* ≤ 0.01 ^†^	*p* ≤ 0.01 ^†^	*p* ≤ 0.01 ^†^
SSI	72	221.71 ± 1.92 ^b^	93.11 ± 1.69 ^b^	2.48 ± 0.03 ^a^	5.25 ± 0.10 ^b^	2.65 ± 0.02 ^a^	6.93 ± 0.13 ^b^	1.35 ± 0.03 ^a^	5.27 ± 0.05 ^a^
UV	72	245.33 ± 1.92 ^a^	101.83 ± 1.69 ^a^	2.47 ± 0.03 ^a^	6.56 ± 0.10 ^a^	2.64 ± 0.02 ^a^	8.29 ± 0.13 ^a^	1.71 ± 0.03 ^b^	5.04 ± 0.05 ^b^
**Catalyst loading [mg/mL]**		*p* ≤ 0.01 ^†^	*p* ≤ 0.01 ^†^	*p* ≤ 0.01 ^†^	*p* ≤ 0.01 ^†^	*p* = 0.15 ^‡^	*p* ≤ 0.01 ^†^	*p* ≤ 0.01 ^†^	*p* = 0.08 ^‡^
0.5	48	221.52 ± 2.35 ^b^	83.73 ± 2.07 ^b^	2.71 ± 0.04 ^a^	5.69 ± 0.13 ^b^	2.63 ± 0.03 ^a^	7.33 ± 0.16 ^b^	1.43 ± 0.04 ^b^	5.27 ± 0.07 ^a^
1.0	48	236.88 ± 2.35 ^a^	102.00 ± 2.07 ^a^	2.39 ± 0.04 ^b^	6.18 ± 0.13 ^a^	2.69 ± 0.03 ^a^	8.00 ± 0.16 ^a^	1.64 ± 0.04 ^a^	5.08 ± 0.07 ^a^
2.0	48	242.17 ± 2.35 ^a^	106.69 ± 2.07 ^a^	2.33 ± 0.04 ^b^	5.83 ± 0.13 ^b^	2.61 ± 0.03 ^a^	7.50 ± 0.16 ^b^	1.52 ± 0.04 ^b^	5.12 ± 0.07 ^a^
**Irradiation time [min]**		*p* ≤ 0.01 ^†^	*p* ≤ 0.01 ^†^	*p* = 0.07 ^‡^	*p* ≤ 0.01 ^†^	*p* ≤ 0.01 ^†^	*p* ≤ 0.01 ^†^	*p* ≤ 0.01 ^†^	*p* = 0.72 ^‡^
30	48	237.79 ± 2.35 ^a^	98.31 ± 2.07 ^a^	2.51 ± 0.04 ^a^	5.76 ± 0.13 ^b^	2.65 ± 0.03 ^b^	7.47 ± 0.16 ^b^	1.49 ± 0.04 ^b^	5.19 ± 0.07 ^a^
60	48	224.21 ± 2.35 ^b^	91.40 ± 2.07 ^b^	2.51 ± 0.04 ^a^	6.31 ± 0.13 ^a^	2.73 ± 0.03 ^a^	8.13 ± 0.16 ^a^	1.62 ± 0.04 ^a^	5.15 ± 0.07 ^a^
120	48	238.56 ± 2.35 ^a^	102.71 ± 2.07 ^a^	2.40 ± 0.04 ^a^	5.63 ± 0.13 ^b^	2.54 ± 0.03 ^c^	7.24 ± 0.16 ^b^	1.48 ± 0.04 ^b^	5.12 ± 0.07 ^a^
**GRAND MEAN**	144	233.52 ± 1.33	97.47 ± 1.20	2.48 ± 0.02	5.90 ± 0.06	2.64 ± 0.02	7.61 ± 0.09	1.54 ± 0.03	5.15 ± 0.04

Results are expressed as mean ± standard error. Values represented with different letters are statistically different at *p* ≤ 0.05; ^†^ significant factor in multifactor analysis; ^‡^ not significant factor in multifactor analysis. m(AG/P)/[mg]—shoot mass (=above-ground mass) per plant; m(R/Plant)/[mg]—root mass per plant; m(AB/R)/[mg]—shoot/root ratio; SSI—simulated solar irradiation; Chl a—Chlorophyll a; Chl b—Chlorophyll b; Chl a+b—sum of chlorophyll a+b; car—carotenoids; Chl a+b/car—sum of chlorophyll a+b/carotenoids ratio.

## Data Availability

Not applicable.
